# [Retracted] LOC102724163 promotes breast cancer cell proliferation and invasion by stimulating MUC19 expression

**DOI:** 10.3892/ol.2024.14747

**Published:** 2024-10-15

**Authors:** Tao Jiang, Zhong-Bing Luo

Oncol Lett 23: 100, 2022; DOI: 10.3892/ol.2022.13220

Following the publication of this paper, it was drawn to the Editor's attention by a concerned reader that certain of the Transwell invasion assay data shown in Fig. 7D on p. 10 were strikingly similar to data that had already been published in different form in another article written by different authors at a different research institute [Jiang X and Chen D: LncRNA FAM181A-AS1 promotes gliomagenesis by sponging miR-129-5p and upregulating ZRANB2. Aging 12: 20069-20084, 2020].

Owing to the fact that the contentious data in the above article had already been published prior to its submission to *Oncology Letters*, the Editor has decided that this paper should be retracted from the Journal. The authors were asked for an explanation to account for these concerns, but the Editorial Office did not receive a reply. The Editor apologizes to the readership for any inconvenience caused.

